# Feasibility Aspects of Exploring Exercise-Induced Neuroplasticity in Parkinson's Disease: A Pilot Randomized Controlled Trial

**DOI:** 10.1155/2020/2410863

**Published:** 2020-03-25

**Authors:** Hanna Johansson, Malin Freidle, Urban Ekman, Ellika Schalling, Breiffni Leavy, Per Svenningsson, Maria Hagströmer, Erika Franzén

**Affiliations:** ^1^Department of Neurobiology, Care Sciences and Society, Division of Physiotherapy, Karolinska Institutet, Stockholm, Sweden; ^2^Medical Unit Occupational Therapy & Physiotherapy, Allied Health Professionals Function, Karolinska University Hospital, Stockholm, Sweden; ^3^Department of Neurobiology, Care Sciences and Society, Division of Clinical Geriatrics, Karolinska Institutet, Stockholm, Sweden; ^4^Department of Clinical Science, Intervention and Technology, Division of Speech and Language Pathology, Karolinska Institutet, Stockholm, Sweden; ^5^Speech Language Pathology, Medical Unit, Karolinska University Hospital, Stockholm, Sweden; ^6^Stockholm Sjukhem Foundation, Stockholm, Sweden; ^7^Department of Clinical Neuroscience, Division of Neurology, Karolinska Institutet, Stockholm, Sweden; ^8^Sophiahemmet University, Department of Health Promoting Science, Stockholm, Sweden

## Abstract

**Background:**

Recent studies indicate that exercise can induce neuroplastic changes in people with Parkinson's disease (PwPD). Reports of feasibility outcomes from existing pilot trials however are, of date, insufficient to enable replication by others in larger definitive trials.

**Objective:**

To evaluate trial design for a definitive trial by exploring process and scientific feasibility.

**Methods:**

The trial design was a parallel-group RCT pilot with a 1 : 1 allocation ratio to either HiBalance or an active control group (HiCommunication). Both groups received one-hour sessions twice weekly, plus home exercises weekly, for 10 weeks. Participants with mild-to-moderate Parkinson's disease (PD) were recruited via advertisement. Assessment included physical performance, structural and functional MRI, blood sampling, neuropsychological assessment, and speech/voice assessment. Process and scientific feasibility were monitored throughout the study. Process feasibility involved recruitment, participant acceptability of assessments and interventions, assessment procedures (focus on imaging, blood sampling, and dual-task gait analysis), and blinding procedures. Scientific feasibility involved trends in outcome response and safety during group training and home exercises. Data are presented in median, minimum, and maximum values. Changes from pre- to postintervention are reported descriptively.

**Results:**

Thirteen participants were included (4 women, mean age 69.7 years), with a recruitment rate of 31%. Attendance rates and follow-up questionnaires indicated that both groups were acceptable to participate. Image quality was acceptable; however, diplopia and/or sleepiness were observed in several participants during MRI. With regard to dual-task gait performance, there appeared to be a ceiling effect of the cognitive tasks with seven participants scoring all correct answers at pretest. Blinding of group allocation was successful for one assessor but was broken for half of participants for the other.

**Conclusions:**

The overall trial design proved feasible to perform, but further strengthening ahead of the definitive RCT is recommended, specifically with respect to MRI setup, cognitive dual-tasks during gait, and blinding procedures. This trial is registered with NCT03213873.

## 1. Background

The benefits of physiotherapy interventions among people with Parkinson's disease (PwPD) have been confirmed [1, 2]. If these improvements in behavior can be specifically linked to brain plasticity, this knowledge could guide clinicians when choosing and progressing programs that are conducive to more enduring changes.

To date, however, insufficient methodological quality and variable results inhibit any firm conclusions to be drawn between how rehabilitation influences the neural networks in Parkinson's disease (PD) [3]. Developing the methods of exploring neuroplastic mechanisms that underlie behavioral improvements following exercise intervention in PD is therefore a current priority within neurorehabilitation research.

Findings from existing publications on exercise-induced neuroplasticity among PwPD indicate a promising possibility of exercise-induced neuroplasticity [4, 5]. However, the clinical studies supporting this evidence are often nonrandomized and small-sampled, and few have progressed from pilot study to full-scale randomized controlled trial (RCT), which are necessary to establish efficacy. It is possible that the gap in studies progressing from pilot to definitive trials is explained by a lack of transparent reporting of feasibility and scientific outcomes of these often complex designs.

We plan therefore to investigate whether a ten-week highly challenging, supervised balance program for PD (HiBalance), with proven efficacy and effectiveness [6, 7], can result in neuroplastic changes. As a progression from previous HiBalance trials, we will incorporate an active control group in the current study design. This feature will add robustness to any potential correlations between improvements in gait and balance and brain plasticity, while also controlling for influential factors such as social support, therapist attention, and length of involvement [8]. This novel study design is previously untested, and this pilot phase is an important step before commencing the definitive trial. Additionally, the transparent reporting of process and scientific outcomes should be of benefit to researchers in the field who wish to replicate feasible components, while also avoiding the repetition of disadvantageous elements. The aim of this pilot study was to systematically evaluate the process and scientific feasibility of a trial design to investigate exercise-induced neuroplasticity of the HiBalance program.

## 2. Methods

A study protocol of the definitive trial, for which this study is an external pilot, was registered with clinicaltrials.gov (NCT03213873). Reporting of the study follows the Consolidated Standards of Reporting Trials (CONSORT) 2010 statement: Extension to randomized pilot and feasibility trials (see Supplementary Materials for checklist). [9] The trial design was a parallel-group RCT pilot with an equal 1 : 1 allocation ratio to either the HiBalance group or the active control group.

### 2.1. Trial Objectives

The process feasibility objectives were as follows:

(1) To assess the number of PwPD reporting interest in participating in the study and to determine whether eligibility criteria were sufficient but not overly restrictive by estimating feasible eligibility and recruitment rate.
(2) To assess compliance with and acceptance of both the HiBalance intervention and the control group intervention by monitoring attendance rates, retention rates, and evaluating participant experiences.
(3) To investigate feasibility of new assessment methods by the following:
  - Evaluating image quality, experimental setup, and participant acceptability of the imaging assessment.
  - Monitoring compliance with blood sampling.
  - Investigating which of two cognitive dual tasks during gait analysis would be most feasible for the definitive trial with regard to performance and experimental setup.
(4) To assess the ability to maintain blinding among assessors.

The scientific feasibility objectives were as follows:

1. To explore trends in outcome measures, including physical performance, well-being, executive function, and voice intensity.
2. To assess safety by monitoring adverse events at both group sessions and during home exercises.

### 2.2. Participants

Participants were recruited via advertisement in the local newspaper and the National Parkinson Association Sweden and through referral from the Parkinson team at the Karolinska University Hospital, Stockholm, Sweden. Potential participants were initially screened via telephone and were thereafter assessed for eligibility in a university setting. Participants were eligible for inclusion if they (1) had a diagnosis of Idiopathic PD, (2) were ≥60 years of age, (3) were Hoehn and Yahr stage 2-3 [10], and (4) scored ≥21 on Montreal Cognitive Assessment (MoCA) [11]. Participants were excluded if they had (1) magnetic resonance imaging (MRI) incompatible implants or claustrophobia or (2) any other neuromuscular disorder that impacted gait and balance function.

### 2.3. Interventions

The training regimens were designed in order to match on as many principles (dose, progression, etc.) as possible without overlapping on respective intervention-targeted behavior. Both interventions were delivered in a group setting with the same dose (10 weeks), frequency (twice per week), and length per session (60 minutes). Details of the training regimens are summarized in Table 1. All participants were advised against commencing any other new training programs during their period of involvement in the study. Participants answered anonymous follow-up questionnaires on their experiences with the interventions after the training period.

#### 2.3.1. HiBalance (Intervention Group)

The methodology and theoretical underpinnings of the HiBalance training have been described in detail elsewhere [12]. In summary, the program consisted of highly challenging balance exercises specifically targeting four core areas of balance control often impaired in PwPD: (1) sensory integration; (2) anticipatory postural adjustments; (3) motor agility, and (4) stability limits. The difficulty level of the training sessions was increased progressively by involving both motor and cognitive dual tasks. Tasks were individually adjusted by trainers in order to ensure that exercises were highly challenging.

#### 2.3.2. HiCommunication (Control Group)

Participants in the control group received a training program for speech and communication. This type of training was chosen because we wanted it to be relevant for PD symptomatology in order for participants to feel as motivated to attend the active control group as the HiBalance group. At the same time, we also did not want the control training to affect balance or gait as we want to be able to relate training effects from both groups to separate neural correlates. The program targeted four core areas of relevance for communication in PwPD: voice intensity, articulatory precision, word retrieval, and memory. Exercises were made increasingly difficult by adding memory challenging tasks, by requiring more communicative interaction between participants, or by adding increased background noise.

#### 2.3.3. Home Exercise Program (HEP)

Home exercise programs were instructed at the first training session and participants were requested to perform these once a week during the training period. The HEP for the HiBalance group focused on functional aerobic and strength exercises, whereas the HEP for the control group focused on exercises for voice and speech function. Both HEPs were instructed to be performed with a progression in the level of difficulty throughout the training period. Participants were also asked to fill out a simple diary in which they stated whether home exercises had been performed (yes/no) each week.

### 2.4. Outcome Assessments

Participants were assessed on three occasions both before and after intervention in their ON state at Karolinska Institutet and Karolinska University Hospital. Each session lasted between 90 and 120 minutes and was performed on separate days in order to minimize the risk of fatigue. Each corresponding session (e.g., physical performance) was performed at approximately the same time of day before and after. All assessments were performed within a period of three weeks both before and after. See Table 2 for assessment schedule. The rationale for collecting this multitude of data relates to the study aim in that it enabled the exploration of feasibility and participant acceptability of the data collection methods.

#### 2.4.1. Physical Performance

Balance was assessed using the Mini-Balance Evaluation Systems Test (Mini-BESTest, a 14-item clinical test covering four components of balance control [13]. Patient-reported balance confidence was captured using the Activities-specific Balance Confidence scale (ABC scale) [14]. Comfortable gait speed was assessed under single- and dual-task conditions using an electronic walkway system (GAITRite®, CIR Systems Inc., PA). In order to investigate the most feasible dual task during gait, two different cognitive tests of executive function were performed: an auditory Stroop task [15] and an N-back task [16] (two-back). Both tasks were performed in auditory versions where stimuli were presented via wireless headphones with a variable interval of 1.5–2.5 seconds in order to control for cueing effects. Participants were instructed to respond to respective stimuli as fast as possible. During auditory Stroop, participants were presented with the Swedish words for “high” and “low” with congruent and incongruent high and low tones and were to respond verbally to the corresponding tone. N-back participants were presented with a string of numbers where they were instructed to answer *yes* when they heard the same number that had been read two numbers back and *no* to all other numbers. Both tasks were also performed in sitting as single tasks. The order of performance on single tasks versus dual tasks and the order of performance of auditory Stroop versus N-back were randomized. Self-reported walk difficulty was captured using the Walk-12 scale [17]. Participants' mean steps per day were assessed using a waist-worn accelerometer (Actigraph GT3X+, Pensacola, FL, USA) for seven consecutive days on two occasions: pre- and postintervention.  Section 3 of the MDS-UPDRS was used to evaluate motor function [18].

#### 2.4.2. Well-Being

Quality of life and health status were assessed using Euroqol 5 dimensions (EQ5D), a widely used generic instrument [19], and Parkinson Disease Questionnaire-39 (PDQ-39), an instrument covering the impact of PD on specific dimensions of functioning and well-being [20]. Symptoms of depression and anxiety were assessed using the Hospital Anxiety and Depression Scale (HADS) [21]. Sections 1-2 of the MDS-UPDRS were used to evaluate motor and nonmotor aspects of everyday life [18].

#### 2.4.3. Brain Imaging

A Philips Ingenia CX 3 Tesla MRI scanner was used. Participants underwent structural MRI (T1 and T2), resting state fMRI as well as two task-based fMRI in the following scan acquisition order: T1, resting state, single task, T2, and dual task. We aimed to investigate the same domains of abilities as in the assessment of physical performance outside the scanner, that is, motor ability measured by a single-task design as well as with a dual-task design. Due to the constraints of movement in an MRI scanner and the deleterious impact of head movements on image quality, we used tasks where participants were to only use their fingers. For the single task, four white circles on a horizontal line were shown on a black screen with a different circle turning grey every 1.2 second. Participants had four buttons and were to press the button corresponding to the circle turning grey. Task outcomes were reaction time and correct response. The dual task was designed as the single task with the addition of a plus sign that intermittently showed up just above the circles. In the dual task, participants were not only to press the button corresponding to the circle turning grey but also to count how many plus signs they had noticed.

Due to several encountered difficulties including participants falling asleep and experiencing severe diplopia, a pure feasibility evaluation of the brain imaging related data was chosen and focused solely on the pretesting. We evaluated the imaging sequences of most importance to the study, T1 and the single task. The single task was of special interest to us since it served as our main proxy measure for motor ability outside the scanner. We also reasoned that the quality of the brain activity data for the single task would be representative for the other functional sequences, that is, the dual task and the resting state. Furthermore, the T1 sequence is a prerequisite for the analysis of fMRI data (to coregister the activity pattern to each participant's brain structure) but also of interest to enable investigation of potential structural brain changes due to the intervention. Overall quality of T1 was evaluated using the Computational Anatomy Toolbox for SPM (CAT12, http://www.neuro.uni-jena.de/cat/) applied to Statistical Parametric Mapping analysis package (SPM12, http://www.fil.ion.ucl.ac.uk/spm/software/spm12/), and for single-task data, we evaluated the degree of movement by framewise displacement using MATLAB (R2015a). The problems encountered at task performance data were summarized. In addition, participant experience of the MRI assessment was investigated with an in-house developed questionnaire gathering data on comfortableness and anxiety and also information that could affect task performance such as tiredness, pain, tremor, and sleepiness.

#### 2.4.4. Blood Sample

Blood samples were collected at both training sites (hospital setting) by a registered nurse prior to first and last training session in order to assess levels of brain-derived neurotrophic factor (BDNF) in plasma.

#### 2.4.5. Cognitive Function

Participants were assessed with a neuropsychological test battery (60–70 minutes) targeting the following cognitive domains: executive function, attention/working memory, episodic memory, and visuospatial functions; see Table 2 for specific tests.

#### 2.4.6. Voice

A standardized speech recording was performed in a sound-treated booth. Mean voice intensity (dBSPL) during text reading was analyzed from the recording to evaluate the effects of intervention in the control group. Dysarthria was assessed according to Hartelius [22] and included speech intelligibility (words and sentences) and a self-report questionnaire on acquired speech disorder [23].

As one of the scientific feasibility objectives was to look at outcome trends, a selected number of the abovementioned assessments will be presented in the result section. Selection was based on the following: the Mini-BESTest as this will be the main outcome for the definitive trial; gait speed and dual-task cost on gait speed as the dual tasks are also part of the process feasibility evaluation; PDQ-39 as this is the only PD-specific included measure pertaining to self-reported health status; tests of executive function as our strongest hypothesis for the neuropsychological assessments relates to this cognitive domain and lastly voice intensity as a representative measure related to the control group intervention.

### 2.5. Sample Size

As we wanted to design and conduct the pilot study in order to support the future development of a definitive trial, we needed enough participants to fill one intervention group and one control group. As established in previous studies, the HiBalance training is conducted in groups of six to eight participants. Aiming at an equal number of participants in the control group, the goal was to include 12–16 participants in total. This sample size does not provide enough power to detect statistically significant effects but will enable both feasibility exploration and guide sample size calculations for the definitive trial. Power calculation for the definitive trial will be based on the Mini-BESTest as this will be the primary outcome.

### 2.6. Randomization

A block randomization (block sizes 2, 4, and 6) was created using a computerized random sequence generator (http://www.randomization.com). Information was placed in sealed, numbered envelopes by a researcher not involved in recruitment, assessment, or training and distributed consecutively after each MRI assessment.

### 2.7. Blinding

Blinded assessors performed pre- and posttesting of physical performance (balance, gait, and motor function), as well as speech, voice, and cognitive function. Blinding was evaluated using a questionnaire inspired by Lowe et al. [24]. The two assessors of physical performance answered whether they were aware or unaware of group allocation by choosing one of five alternatives: (1) I do not know which group the participant is in, (2) I have guessed that the participant is in the HiBalance group, (3) I have guessed that the participant is in the HiCommunication group, (4) the participant has told me they are in the HiBalance group, or (5) the participant has told me they are in the HiCommunication group.

### 2.8. Statistical Analysis

Data were analyzed using IBM SPSS Statistics for Macintosh, Version 25.0 (Armonk, NY: IBM Corp.). Due to the small sample size, all data are presented in median values along with minimum and maximum values. Dual-task cost on gait speed was calculated as the absolute difference between dual-task and single-task conditions and expressed in percent ((dual-task − single-task)/singe-task) × 100). Changes from pre- to postintervention are reported descriptively. Change expressed as median difference was analyzed by calculating change variables (postvalue minus prevalue).

## 3. Results

### 3.1. Participant Recruitment

**Figure 1:**
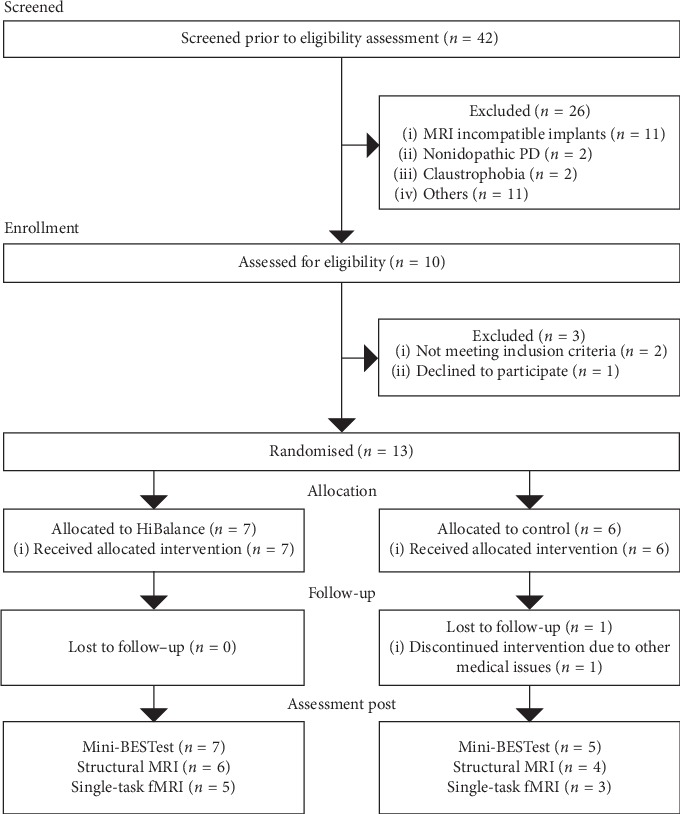
Consort flow diagram of participants.

Recruitment began in May 2017 and data collection concluded in December 2017. Forty people responded to the advertisement, and two people were approached after referral from the Parkinson team at the Karolinska University Hospital. After initial telephone screening for exclusion criteria, 16 of these potential participants were assessed for eligibility. Fourteen participants were eligible for inclusion, but one person chose to withdraw prior to randomization, rendering a total recruitment rate of 31% (13 out of 42). See Figure 1 for flow diagram. Demographics of participants in both groups are summarized in Table 3. One exclusion criterion, *inability to hear instructions without a hearing aid*, was added during the course of the study as this would have hindered communication during the MRI session.

### 3.2. Interventions

Mean overall attendance rate was 84.3% in the HiBalance group and 89.0% in the control group. One person in the control group dropped out after nine training sessions, due to medical reasons unrelated to the intervention or PD. All participants in both groups (one missing in the HiBalance group) reported having performed the HEP throughout the training period. Two out of six respondents in the HiBalance group stated having progressed the intensity and/or complexity of exercises as recommended. In the control group, participants were instructed new and gradually more complex home exercises each week, thereby ensuring progression given that they performed the HEP. All participants, irrespective of group allocation, stated that they would recommend this type of training to other PwPD. There were two noninjurious falls during group training in the HiBalance group. One participant also reported two noninjurious falls when performing the HEP. Feelings of increased tiredness in connection to the group training were reported to a low degree by one participant, to some extent by three participants, and to a high degree by one participant in the HiBalance group. Participants did not report a cessation of other activities due to tiredness, pain, or other symptoms. In the control group, three participants reported no feelings of increased tiredness, while two reported feeling this to some extent.

### 3.3. Outcome Assessments

Below are process and scientific feasibility reported for each outcome domain. Outcome trends reported on prespecified tests and questionnaires (see Section 2) are presented in Table 4, whereas absolute values on all outcome measures are presented in Supplementary Materials.

#### 3.3.1. Physical Performance and Well-Being

**Figure 2:**
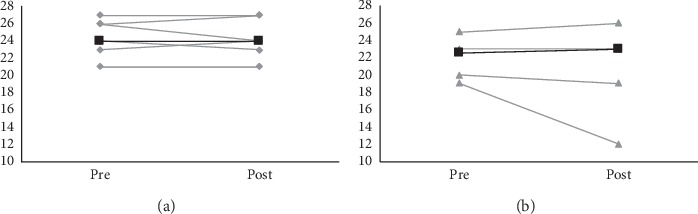
Absolute values on the Mini-BEST test of individuals (grey lines) and group median values (black lines) for both groups at pre- and postassessment. (a) HiBalance group. (b) Control group.

With regard to experimental setup and participant comprehension of instructions, the auditory Stroop task proved more feasible to perform as a dual-task during gait compared to N-back. However, there was a ceiling effect with seven participants scoring all correct answers at pretest. After the intervention period, the HiBalance group performed similarly on dual-task gait, with median differences in cost on gait speed of 1.9% and 3.0% on N-back and auditory Stroop, respectively. The control group showed a small tendency to improve on both tasks (3.8% and 6.4%, resp.). As for the Mini-BESTest, results showed no median difference after intervention in either group; see Figure 2 for individual scores before and after intervention. Trends on self-reported health status (PDQ-39 summary index) showed an improvement by 8.4% in the HiBalance group, while remaining close to be unchanged (1.7% decrease) in the control group.

#### 3.3.2. Brain Imaging

There were several problems encountered during our MRI paradigm. First, despite rigorous screening procedures, two of the 13 included participants could not undergo the MRI because of uncertainties with anamnestic information of metal splinters in the eyes that was not reported until right before the scanning session. In addition, one participant was unable to perform any of the task fMRI since the mirror showing the tasks could not be fitted. This was due to increased slope of the head coil because of kyphosis. At the pretesting, four participants experienced diplopia or other vision problems affecting their performance during the tasks. Three participants fell asleep during one or both of the tasks fMRI and four during resting state fMRI. Two performed the single task at chance level and one had great difficulties. All in all, only four participants allocated to the HiBalance group and three participants in the control group had acceptable behavioral data at the pretesting session. Given the range of problems profoundly affecting the data during the MRI session, it was not deemed sensible to make any analyses of the data but very basic quality assessments. As for the structural data, the overall quality of the T1 images was deemed to be of acceptable or good quality. The head motion of the single-task data was assessed by framewise displacement. Mean framewise displacement ranged from 0.09 to 0.31 mm, four participants had more than 15% spikes above 0.5 mm, and one had a single spike just above 2 mm. Overall, this was deemed as an acceptable level of head motion.

As for participant experience, three participants found the assessment physically uncomfortable and five experienced some emotional unpleasantness. One participant deemed the single task as too difficult and four deemed the dual-task as too difficult.

#### 3.3.3. Blood Sampling

One person declined to leave a blood sample due to fear of needles, but compliance with blood sampling was otherwise good. Due to logistical shortcomings and the handling process, a multitude of the blood samples was missing, and no absolute value data on BDNF will therefore be presented in this sample.

#### 3.3.4. Cognitive Function

Tests of executive function showed a trend toward improvement in the HiBalance group on two measures (switches on semantic verbal fluency and color word interference trial IV), whereas the control group showed trends toward improvement on three measures (trail making test trial IV, switches on semantic verbal fluency, and color word interference trial III).

#### 3.3.5. Voice Intensity

Sound pressure levels indicated no change after intervention in the HiBalance group, whereas there was a median difference increase by 2.5 dBC in the control group.

### 3.4. Blinding

At postassessment, blinding of group allocation was successful in the majority of participants for assessor 1 (10 out of 12), but only in half (6 out of 12) for assessor 2. All occasions with broken blinding contained “I have guessed” responses. All guesses were established to be correct.

## 4. Discussion

This pilot study examined a proposed RCT design with regard to process and scientific feasibility. According to the results, the overall trial design was found to be feasible and acceptable and thus suitable for the definitive trial.

### 4.1. Recruitment

One-third of people who reported interest in participating were eligible for inclusion, and the majority of exclusions were based on MRI-incompatible implants. Despite a rigorous screening protocol, two participants with a history of metal splinters in their eyes were, however, included as this was not reported until right before the scanning session. We were unable to scan these participants, but they remained in the study. Given this, an even more rigorous screening of MR incompatible circumstances at first telephone contact for the future RCT is recommended.

### 4.2. Acceptability and Safety of the Interventions

Attendance rates and follow-up questionnaires suggest that both the HiBalance intervention and control group intervention were acceptable to participants. The low number of adverse events further indicates that both interventions are safe for the definitive trial. However, although all participants stated having performed the HEP, few participants in the HiBalance group had progressed the home exercises as recommended. Whether this was due to lack of information or poor compliance remains unclear. Nonetheless, these observations highlight the necessity for trainers to allocate more time for home exercise instruction at first training session. Trainers should also allocate time half-way into the training period to instruct participants about exercise progression. Furthermore, the home exercise diary will need modification, and information regarding exercise progression will be added.

Participants in the HiBalance group reported feeling increased tiredness in relation to the group training, some to a low and some to a high degree. However, this did not impact their everyday life in that they did not report the cessation of any other activities. Since the HiBalance program is a training regimen that progressively challenges both motor and cognitive abilities, feelings of tiredness are therefore to be expected.

### 4.3. Acceptability and Feasibility of New Data Collection Methods

Overall, participants tolerated the MRI assessments well. However, issues related to diplopia, sleepiness, and the difficulty level of the tasks motivate several modifications for the definitive trial. In addition to modifying the task paradigm, we will also perform another pilot study on the new versions of the tasks before implementing them in the definitive trial. Visual symptoms are common in PD [25], and among nondemented PwPD, double vision is experienced by around one-fifth [26]. The drowsiness that several of our participants experienced is also an impairing factor [25]. To counteract this in the definitive trial, we will as far as possible perform the imaging paradigm at a time of day where the participant experiences the least daytime somnolence. In the pilot study to be performed, we will also evaluate whether modifications of the presentation of the visual stimuli in the tasks can ease straining of the eyes as well as diplopia. In addition, to further diminish the risk of tiredness and strained eyes, we will evaluate whether shorter versions of the tasks are feasible. Lastly, we will do a rearrangement of the scanning sequences such that those of most importance will be placed earlier on in the assessment. The new scan acquisition order will be as follows: single task, T1, dual task, resting state, and T2.

The auditory Stroop task proved superior to N-back as a dual task during gait with regard to the experimental setup, that is, intermittent gait, and comprehension of instructions among participants. However, given that several of the participants scored correct on all stimuli, the level of difficulty needs to be adjusted, such as decreasing the length of the interstimulus intervals, in order to lower the risk of a ceiling effect in the definitive trial.

When studying circulating levels of BDNF, it has been suggested that serum is more appropriate than plasma. It has also been debated whether BDNF levels in plasma and serum can be compared [27]. Given these arguments, it seems reasonable that, for a definitive trial, the method should be changed from plasma to serum. More importantly, we experienced numerous missing data due to logistical issues related to strained clinical resources. A more stringent and streamlined handling of blood samples is therefore required in order for serum samples to be incorporated in a larger trial running at two geographical sites. In light of this, a core facility will be engaged in the project, ensuring that all samples will be collected and preprocessed in the exact same manner, while also securing all samples to be stored safely in the same designated place.

### 4.4. Blinding

The blinding process needs to be more rigorous at all stages for the definitive trial. Strengthening of the blinding procedures such as providing writing and verbal clarification to participants about keeping group allocation secret will be required. It is also advisable that all assessors should be blinded and asked to fill out blinding questionnaires after intervention. Another important variable to consider in clinical trials is to what extent participants expect the intervention to improve their symptoms [28]. In this pilot trial, participants were not prompted on their expectancy regarding their allocated intervention. Adjustments to the study design for the definitive trial, such as adding an expectancy questionnaire, could amend this and thereby help reduce the risk of bias related to expectancy.

### 4.5. Trends in Treatment Response

Although we primarily focused on exploring feasibility, we did also investigate trends in treatment response on physical performance, well-being, cognitive function, and voice intensity. Balance performance as measured with the Mini-BESTest showed no trends towards effect in either group. While seemingly disconcerting, we do however maintain our confidence in the HiBalance intervention, given the positive results in previous large-scale trials [6, 29]. Comfortable gait speed showed a tendency to increase more in the HiBalance group as compared to the control group, and this change (0.05 m/s) was close to the clinically meaningful difference of 0.06 m/s [30]. Results in dual-task costs on gait speed are somewhat more ambiguous, with trends showing slight improvements in the control group. Baseline characteristics indicate a certain between-group imbalance. Given the small sample size, such imbalance is more likely, but nonetheless needs to be considered when interpreting these results. The drop-out in the control group may also have skewed the median at post-assessments in a false-positive direction.

Health-related quality of life as measured with PDQ-39 showed positive trends in the HiBalance group compared to the control group. As previous meta-analysis indicates, exercise may improve QoL in PwPD [31, 32], and since the HiBalance group targets physical fitness more than the control group, this may explain our results. Another explanation could be that the number of items pertaining to each subdomain in the PDQ-39 is not equally distributed. For example, the number of items concerning mobility is 10, while the number of items concerning communication is three, which may lead to PDQ-39 not being sensitive enough to mirror communication-related quality of life.

Both groups incorporated exercises challenging cognitive abilities. With regard to executive function, the results are mixed with some tests in favor of the HiBalance group and some in favor of the control group. Both groups, however, increased the number of switches during semantic verbal fluency, signaling an improved cognitive flexibility. However, the small sample size makes it difficult to draw firm conclusions. Voice intensity measures showed that control group participants improved, while the HiBalance group did not which indicates that the components of the control group were active for the intervention-targeted behavior.

### 4.6. Strengths and Limitations

The greatest strength of this study is the rigorous methodology exploring both process and scientific feasibility while also including a multitude of outcome assessments with the intention of optimizing the protocol of a definitive trial. Another strength is that intervention and control group training regimens have been designed to be equivalent on as many elements as possible, thereby enabling factors such as social support to be controlled for [8]. Using an active control group also enables a more realistic exploration of recruitment and retention rates, as well as randomization and blinding processes [33]. We do however acknowledge a number of limitations of the study. One is the dilemma of power calculation for the definitive trial. Given that we intend to detect how change in behavior relates to neuroplasticity, it would be optimal if we could conduct a power calculation for this association. Since this is not possible, we decided to conduct the power calculation on the primary outcome, in this case balance performance (the Mini-BESTest). Even though the Mini-BESTest did not show trends for improvement within the current sample, our previous larger scale trials have shown this test to be sensitive to changes in populations with mild-to-moderate PD [6, 7].

Another limitation is that the small sample size limits our ability to draw conclusions from outcome assessments. With that in mind, however, it is the results of the process feasibility evaluation that will inform and refine the design of the definitive trial. Having conducted this pilot trial to investigate feasibility of the planned design has been crucial for development of the larger trial. However, we believe that knowledge acquired may also provide key information to other researchers in the planning stages of similar studies among PwPD or other neurological diseases.

## 5. Conclusions

This study demonstrated that our design evaluating the effects of highly challenging balance training with various markers of physical and neuropsychological abilities, as well as brain structure and function, is acceptable from a patient perspective. We are however aware that a total of three assessment sessions both before and after intervention may have exceeded what volunteer participants tolerate and also create a logistical challenge to coordinate the study and require considerable resources. Overall, the different elements of the design were feasible. However, ahead of the definitive trial, we recommend modifications specifically with respect to blinding procedures and expectancy as well to the MRI paradigm and the dual-task gait assessment.

## Figures and Tables

**Table 1:** Descriptions of intervention and control group.

Detail	HiBalance, intervention group	HiCommunication, control group
Setting	Exercise hall	Room for group treatment

Personnel	Two physical therapists	One speech and language pathologist

Position performed	Standing and walking	Sitting

Core areas	(i) Sensory integration	(i) Voice intensity
	(ii) Anticipatory postural adjustments	(ii) Articulatory precision
	(iii) Motor agility	(iii) Word retrieval
	(iv) Stability limits	(iv) Memory

Block A, weeks 1-2	Learning exercises, focus on quality. Single-task performance of exercises pertaining to each core area	Learning exercises, improving speech technique by practicing breathing, phonation, articulation, and increased vocal loudness while maintaining good voice quality

Block B, weeks 3–6	Increased difficulty of exercises by adding cognitive and motor dual tasks	Increased difficulty of exercises by, for example, using memory games and association tasks to increase cognitive load during speech exercises

Block C, weeks 7–10	Complexity increased by combining exercises from all four focus areas and by switching between cognitive and motor dual tasks	Complexity increased by increasing difficulty of memory games, incorporating more interaction between participants, and by adding background noise

Home exercise program, performed once a week	Aerobic capacity (e.g., walking or exercise bike) Leg and core strength exercises	Relaxation and breathing exercises Voice and speech exercises Word and memory exercises

**Table 2:** Assessment schedule.

Outcome domain	Test	Preintervention sessions				Postintervention sessions			
		I	II	III	Others	I	II	III	Others
Balance	Mini-BESTest	√				√			
	ABC-scale	√				√			

Gait	GAITRite® analysis	√				√			
	Walk 12	√				√			

Motor function	MDS-UPDRS part I-III	√				√			

Physical activity	Accelerometer wear, 7 days				√				√

QoL and health status	EQ5D	√				√			
	PDQ-39	√				√			
	HADS	√				√			

Global cognition	MoCA	√				√			

Executive function	Trail making test, trial IV; the color-word interference test; verbal fluency		√				√		
			√				√		
			√				√		
			√				√		

Attention/working memory	Digit span; trail making test, trials I-III		√				√		
			√				√		

Episodic memory	Brief visuospatial memory test; brief visuospatial memory test-revised (BVMT-R)		√				√		

Visuospatial functions	Copy condition from BVMT-R		√				√		

Brain structure and function	Magnetic resonance imaging (MRI)			√				√	
	Task functional MRI (fMRI)			√				√	
	Resting state fMRI			√				√	

Level of BDNF	Blood sample				√				√

Voice intensity	Speech recording		√				√		

Dysarthria	Dysarthria assessment		√				√		

Acceptability	MRI questionnaire			√					
	Intervention follow-up questionnaire								√

Abbreviations: Mini-BESTest, Mini-Balance Evaluation Systems Test; ABC-scale, Activities-specific Balance Confidence scale; MDS-UPDRS, Movement Disorder Society–Unified Parkinsons Disease Rating Scale; EQ5D, EuroQol 5 dimensions; PDQ-39, Parkinsons Disease Questionnaire -39; HADS, Hospital Anxiety and Depression Scale; MoCA, Montreal Cognitive Assessment.

**Table 3:** Demographic and clinical characteristics of the participants at baseline.

Characteristics Median (min–max) unless otherwise stated	HiBalance (*n* = 7)	Control (*n* = 6)
Age, *y*	72.0 (60–78)	67.5 (63–70)
Sex, *n* female	1	3
LEDD (mg)^1^	700 (380–920)	765.5 (525–1171)
Body mass index, kg/m^2^	23.5 (19.6–25.9)	24.4 (21.2–26.8)
Years with PD	10.0 (3–13)	7.0 (3–11)
Hoehn and Yahr^2^, 0–5	2	2.5
2, *n*	5	3
3, *n*	2	3
MDS-UPDRS part III, 0–132^3^	35.0 (24–46)	32.5 (22–52)
Falls in last year, *n*	0 (0–4)	0 (0–3)
Mini-BESTest, 0–28^4^	24.0 (21–27)	22.5 (19–25)
Montreal Cognitive Assessment^5^	27.0 (26–30)	26.5 (21–28)

^1^Levodopa daily equivalent dosage. ^2^Stages of disease progression from 1 to 5 (1 = minimal disability; 5 = confined to bed/wheelchair). ^3^Movevement disorder society-unified Parkinson's disease rating scale, motor examination, where lower scores indicate better motor function. ^4^A 14-item clinical test of balance function (maximum score = 28), where higher scores indicate better balance function. ^5^Cognitive screening-test scoring from 0 to 30, where higher scores indicate better global cognitive function.

**Table 4:** Trends in outcome response.

Outcome measures Median difference (min–max) from pre- to postintervention	HiBalance	Direction	Control	Direction
Physical performance and well-being				
Mini-BESTest, 0-28^a^	0 (−2 to 1)	0	0 (−7 to 1)	0
Comfortable gait speed, m/sec	0.05 (−0.20 to 0.15)	+	0.00 (−0.06 to 0.10)	0
Dual-task cost on gait speed^b^, N-Back, %	1.9 (−9.3 to 22.3)	−	−3.8 (−12.1 to 8.7)	+
Dual-task cost on gait speed^b^, audiostroop, %	−3.0 (−17.7 to 28.6)	+	−6.4 (−26.6 to 8.3)	+
PDQ-39 summary index, %^c^	−8.4 (−32.7 to 1.9)	+	1.7 (−3.5 to 17.4)	−

Executive function				
Trail making test, trial 4, sec^d^	1.0 (−112.0 to 20.0)	−	−28.0 (−82.0 to 16.0)	+
Color word interference, trial 3, sec^d^	2.0 (−7.0 to 17.0)	−	−2.0 (−9.0 to 11.0)	−
Color word interference, trial 4, sec^d^	−7.0 (−36.0 to 16.0)	+	5.0 (−37.0 to 14.0)	−
Verbal fluency, *n* switches^e^	2.0 (0.0 to 3.0)	+	1.0 (−3.0 to 6.0)	+

Voice				
Sound pressure level, dB (C)	0.0 (−1.7 to 2.3)	0	2.5 (−1.3 to 5.6)	+

Direction indicates whether trend in change from pre- to postintervention was positive (+), negative (−), or unchanged (0). ^a^A 14-item clinical test of balance function (maximum score = 28), where higher scores indicate better balance function. ^b^Dual-task cost on gait speed, where negative values indicate trend to improve and positive values indicate trend to decline. ^c^PDQ-39: Parkinson's Disease Questionnaire-39, consisting of eight subdomains and a summary index, scale between 0 and 100. Summary index scale where 0 indicates perfect health as assessed by the measure and 100 indicates worst health as assessed by the measure. ^d^From Delis Kaplan Executive Function System™, negative values indicate trend to improve and positive values indicate trend to decline. ^e^From Delis Kaplan Executive Function System™, positive values indicate trend to improve and negative values indicate trend to decline.

## Data Availability

The datasets generated and/or analyzed during the current study are not publicly available due to Swedish legislation. Data are available upon reasonable request, and requests for data access can be put to our Research Data Office (rdo@ki.se) at Karolinska Institutet and will be handled according to the relevant legislation.

## Abbreviations

PD: Parkinson's disease
PwPD: People with Parkinson's disease
MoCA: Montreal Cognitive Assessment
MRI: Magnetic resonance imaging
HEP: Home exercise program
Mini-BESTest: Mini-Balance Evaluation Systems Test
ABC-scale: Activities-specific balance confidence scale
MDS-UPDRS: Movement disorder society-unified Parkinson's disease rating scale
EQ5D: EuroQol 5 dimensions
PDQ-39: Parkinson's Disease Questionnaire-39
HADS: Hospital Anxiety and Depression Scale
BVMT: Brief visuospatial memory test
fMRI: Functional MRI
SPL: Sound pressure level
BDNF: Brain-derived neurotrophic factor.

## Supplementary Materials

Supplementary Materials Supplementary Material File 1: absolute values on all tests at pre- and postassessments.
