# Supplementary material for: Feasibility Aspects of Exploring Exercise-Induced Neuroplasticity in Parkinson's Disease: A Pilot Randomized Controlled Trial
Source: Parkinsons Dis. 2020 Mar 25;2020:2410863. doi: 10.1155/2020/2410863 (PMC7132585; doi:10.1155/2020/2410863)
Supplement: Supplementary Materials — Supplementary Material File 1: absolute values on all tests at pre- and postassessments. [file 2410863.f1.pdf]

Supplementary Material File 1

Absolute values on all tests at pre and post assessments

**Outcome** median (min-max)	**HiBalance**		**Control**	
**Physical performance tests**	**Pre (n=7)**	**Post (n=7)**	**Pre (n=6)**	**Post (n=5)**
Mini-BESTest [a], 0-28	24.0 (21-27)	24.0 (21-27)	22.5 (19-25)	23.0 (12-26)
MDS-UPDRS[b] III, 0-132	35.0 (24-46)	35.0 (25-44)	32.5(22-52)	35.0 (22-56)
Usual gait speed, m/s	1.4 (1.2-1.6)	1.4(1.3-1.6)	1.3 (0.9-1.5)	1.3 (0.9-1.5)
Gait speed, DT N-back, m/s	1.4 (0.9-1.5)	1.2 (0.9-1.6)	1.0 (0.6-1.4)	1.1 (0.5-1.4)
DT cost N-Back, %	7.2 (1.7 -27.2)	8.9 (6.3-30.2)	18.2(7.0 -35.8)	11.8(5.6-36.3)
Gait speed, DT Audiostroop, m/s	1.4 (0.9-1.6)	1.4 (0.8-1.5)	1.0 (0.5-1.5)	1.2(0.7-1.5)
DT cost Audiostroop, %	3.8 (-0.5-31.7)	5.8 (-4.2-35.5)	15.0 (-5.0-44.6)	7.5 (3.3-23.0)
Mean steps per day (control post n=3)	4256 (2087-10764)	4405 (1337-5904)	5534 (1171-9597)	6582 (6389-8333)
**Self-reported measures**				
ABC scale[c], %	85.0 (57.5-93.1)	90.0 (68.1-94.4)	74.7 (23.8-94.4)	71.3 (58.6-95.6)
Walk 12[d], 0-42	9.0 (4.0-16.0)	4.0 (0-12)	17.0 (1-26)	15.0 (1-16)
PDQ -39[e] Summary Index, 0-100	20.5 (2.9-38.4)	11.8 (3.7-26.8)	20.5 (12.9-30.4)	24.7(11.6-47.8)
HADS[f] anxiety, 0-21	7.0 (3-11)	6.0 (1-9)	5.0 (0-8)	6.0 (4-13)
HADS[f] depression, 0-21	3.0 (1-6)	3.0 (1-7)	2.0 (1-6)	4.0 (3-8)
EQ5D[g] index, -.594-1	0.7 (0.7-0.8)	0.8 (0.2-1.0)	0.8 (0.7-0.9)	0.8 (0.7-0.9)
EQ VAS[h], 0-100	75.0 (60-80)	70.0 (50-95)	72.5 (55-80)	75 (45-85)
MDS-UPDRS[b] I, 0-52	10.0 (3-16)	7.0 (5-21)	7.5 (5-15)	9.0 (4-15)
MDS-UPDRS[b] II, 0-52	15.0 (5-19)	9.0 (2-17)	13.0 (3-20)	12.0 (4-18)
**Neuropshycological test battery**				
Trail Making Test[i] I, 0-150 sec	25.0 (18-32)	29.0 (15-38)	31.5 (20-55)	32.0 (19-38)
Trail Making Test[i] II, 0-150 sec	62.0 (24-323)	39.0 (30-150)	45.0 (25-126)	68.0 (23-78)
Trail Making Test[i] III, 0-150 sec	46.0 (37-99)	36.0 (27-99)	54.0 (27-143)	76.0 (19-115)
Trail Making Test[i] IV, 0-240 sec	154.0 (64-240)	95.0 (68-207)	130.0 (88-240)	143 (65-210)
Trail Making Test[i] V, 0-150 sec	38.0 (26-150)	37.0 (25-97)	56.0 (37-129)	54.0 (22-122)
Color-Word Interference[j] I, 0-90 sec	33.0 (25-38)	32.0 (27-36)	40.5 (33-50)	34.0 (32-45)
Color-Word Interference[j] II, 0-90 sec	25.0 (18-30)	24.0 (22-30)	28 (23-53)	28.0 (23-32)
Color-Word Interference[j] III, 0-180 sec	63.0 (54-77)	64.0 (54-94)	60.0 (52-153)	62.0 (50-164)
Color-Word Interference[j] IV, 0-180 sec	72.0 (57-144)	68.0 (50-128)	73.0 (65-156)	82 (65-128)
VF[k], letter fluency, n words	48.0 (17-77)	50.0 (21-81)	38.0 (29-56)	35 (28-50)
VF[k], semantic fluency, n words	47.0 (25-57)	41.0 (25-53)	38.0 (22-43)	33.0 (22-39)
VF[k], semantic switching, n words	12.0 (10-16)	14.5 (11-19)	10.5 (4-15)	11.0 (7-14)
VF[k], semantic switching, n switches	14.0 (9-16)	14.5 (10-18)	9.5 (3-14)	10.0 (7-13)
BVMT[l] total	23.0 (8-28)	23.0 (12-33)	19.0 (6-31)	13.0 (8-29)
BVMT[l] delayed recall	10.0 (1-12)	8.0 (6-11)	9.0 (2-12)	7.0 (3-12)
Digit Span[m] forward	9.0 (5-12)	9.0 (6-11)	11.0 (6-14)	10.0 (8-13)
Digit span[m] backward	6.0 (5-10)	8.0 (6-12)	7.0 (6-10)	7 (5-9)
RAVLT[n] total	36.0 (29-64)	42.0 (29-63)	33.0 (23-48)	40.0 (37-51)
RAVLT[n] delayed recall	8.0 (4-10)	9.0 (3-14)	7.5 (0-8)	9.0 (1-10)
**Speech and language assessment**				
Sound Pressure Level, dB(C)	70.7 (68.1-75.3)	69.8 (64.8-75.0)	71.1 (68.0-75.3)	73.6 (67.2-76.7)

Dyartria[p] mean score, 0-3	0.06 (0.01-0.60)	0.04 (0.00-0.09)	0.06 (0.02-0.43)	0.02 (0.00.0.18)
Questionnaire on acquired speech disorder[q], mean score, 0-3	0.61 (0.00.0.83)	0.53 (0.05-0.97)	0.61 (0.30-1.30)	0.59 (0.28-1.42)
Word intelligibility, %	98.5 (54.0-100)	98.0 (78.0-100)	95.1 (89.0-100)	98.0 (97.0-100)
Sentence intelligibility, %	100 (75.0-100)	100 (83.0-100)	100 (94.0-100)	100 (98.0-100)

[a]A 14-item clinical test of balance function (maximum score=28), where higher scores indicate better balance function.
[b]Movement Disorder Society – Unified Parkinsons Disease Rating Scale; section I: Non-Motor Aspects of Experiences of Daily Living (0-52), section II: Motor Aspects of Experiences of Daily Living (0-52) , section III: Motor Examination (0-132). In all sections, a higher score indicates greater impairment.
[c] Activities-specific Balance Confidence scale, higher percentage indicates higher balance confidence
[d] Questionnaire on perceived walking difficulty scoring between 0-42 where higher scores indicate a greater impact on walking ability.
[e] Parkinsons Disease Questionnaire -39. Consists of eight subdomains and a summary Index. Scale between 0-100. Summary index scale where 0 indicates perfect health as assessed by the measure and 100 indicates worst health as assessed by the measure.
[f] Hospital Anxiety and Depression Scale, ranges from 0-21 with higher scores indicating higher levels of anxiety or depression respectively.
[g] EuroQol 5 Dimensions health status index varying between 1 and -.594, with 1 being interpreted as complete health and scores below 0 valued as health status worse than dead.
[h] EuroQol Visual Analogue Scale, score between 0 (worst imaginable health) and 100 (best imaginable health)
[I] Trail Making Test from Delis Kaplan Executive Function System™
[j] Color-Word Interference Test, from Delis Kaplan Executive Function System™
[k] Verbal fluency, from Delis Kaplan Executive Function System™
[l] Brief Visuospatial Memory Test
[m] Digit span from Wechsler Adult Intelligence Scale® - fourth edition, Swedish version
[n] Rey Auditory Verbal Learning Test, version 1.
[o]Brain-derived neurotrophic factor
[p] Mean score on dysarthria assessment according to Hartelius et al, with 0 meaning normal function and 3 meaning severe deviation or no function.
[q] Self-report questionnaire containing 30 statements with possible answers ranging from 0 (definitely false) to 3 (definitely true)
